# Edaravone protects against hyperosmolarity-induced oxidative stress and apoptosis in primary human corneal epithelial cells

**DOI:** 10.1371/journal.pone.0174437

**Published:** 2017-03-27

**Authors:** Yanwei Li, Haifeng Liu, Wei Zeng, Jing Wei

**Affiliations:** 1 Department of Ophthalmology, The First Affiliated Hospital and College of Clinical Medicine of Henan University of Science and Technology, Luoyang, China; 2 Department of Ophthalmology, The Third Affiliated Hospital of Xinxiang Medical University, Xinxiang, China; 3 Department of Otorhinolaryngology Head and Neck Surgery, The First Affiliated Hospital and College of Clinical Medicine of Henan University of Science and Technology, Luoyang, China; Texas Technical University Health Sciences Center, UNITED STATES

## Abstract

An increase in the osmolarity of tears induced by excessive evaporation of the aqueous tear phase is a major pathological mechanism behind dry eye. Exposure of epithelial cells on the surface of the human eye to hyperosmolarity leads to oxidative stress, mitochondrial dysfunction, and apoptosis. Edaravone, a hydroxyl radical scavenging agent, is clinically used to reduce neuronal damage following ischemic stroke. In this study, we found that treatment with hyperosmotic media at 400 and 450 mOsM increased the levels of ROS and mitochondrial oxidative damage, which were ameliorated by edaravone treatment in a dose-dependent manner. We also found that edaravone could improve mitochondrial function in HCEpiCs by increasing the levels of ATP and mitochondrial membrane potential. MTT and LDH assays indicated that edaravone could attenuate hyperosmolarity-induced cell death. It was found that edaravone prevented apoptosis by decreasing the level of cleaved caspase-3, and attenuating the release of cytochrome C. Mechanistically, we found that edaravone augmented the expression of Nrf2 and its target genes, such as HO-1, GPx-1, and GCLC.

## Introduction

As a multifactorial disease of the tears and ocular surface, dry eye affects more than 20% of the adult population worldwide. This condition is characterized by inadequacy of the tear film that protects the eyes, thereby having an adverse effect on eye health and comfort [1]. An increase in tear osmolarity induced by increased evaporation of the aqueous tear phase has been considered as a major pathological mechanism behind dry eye. Previous *in vivo* and *in vitro* studies have shown that hyperosmolarity initiates inflammation of the ocular surface and cellular apoptosis in dry eye patients, dry eye mouse models, and in in vitro hyperosmotic culture models of human corneal epithelial cells (HCECs) [2]. Notably, increased levels of ROS, lipid oxidative stress markers, and inflammatory cells have been found in the conjunctiva and tear film of Sjögren’s syndrome patients, suggesting that oxidative stress plays a critical role in the pathogenesis of dry eye [3, 4]. In addition, hyperosmolarity has been recognized as a pro-inflammatory stress factor for the corneal epithelium. Hyperosmolarity leads to an increase in the activation of mitogen-activated protein kinases (MAPKs), such as c-Jun N-terminal kinase (JNK) and p38, which are serine/threonine-specific protein kinases that respond to extracellular stimuli and regulate various cellular activities in the corneal epithelium [5, 6]. Importantly, exposure of epithelial cells on the surface of the human eye to hyperosmolarity leads to apoptosis [7]. Preventing hyperosmolarity-mediated toxicity has become an important therapeutic strategy for the treatment of dry eye.

Edaravone, a hydroxyl radical scavenging agent, is clinically used to reduce neuronal damage following ischemic stroke [8]. Studies have shown that edaravone has a hydroxyl radical (•OH) quenching effect and an inhibitory effect against peroxynitrite (ONOO−) as well as both water-soluble and lipid-soluble peroxyl radicals (LOO•) [9]. In addition to stroke, edaravone has been tested in studies of various diseases, including both neurologic and non-neurologic diseases [10]. Both *in vivo* and *in vitro* experiments have shown that edaravone may be a useful therapeutic tool for early atherosclerosis, pending its clinical efficacy [11]. Based on this idea, we hypothesized that edaravone would inhibit hyperosmolarity-induced oxidative stress and apoptosis in the corneal epithelium. To test this hypothesis, we investigated the effects of edaravone in hyperosmolarity-induced toxicity in the corneal epithelium.

## Materials and methods

### Cell culture and treatment

Primary human corneal epithelial cells (HCEpiCs) were obtained from Life Technologies (USA). Cells were maintained in EpiLife medium supplemented with HCGS and antibiotic (100 μg/ml penicillin–streptomycin) at 37°C in a humidified incubator with 5% CO_2_. Primary HCEpiCs were pretreated with 10 μM or 20 μM of edaravone for 24 h, followed by exposure to hyperosmotic media (400–450 mOsM) for another 24 h.

### Mitochondrial Membrane Potential (MMP)

We assessed the mitochondrial membrane potential (ψm) using the cell permeant, cationic fluorescent dye tetramethylrhodamine methyl ester (TMRM). Cells were washed with HBSS, and then loaded with 20 μM TMRM and incubated for 20 min in darkness to facilitate fluorophore loading. Cells were then washed 3 times with HBSS in darkness. TMRM fluorescence was measured at 548 nm (excitation) and 573 nm (emission). Fluorescence images were analyzed using Image-Pro Plus software to index MMP.

### Measurement of intracellular ROS

We assessed the concentration of intracellular reactive oxygen species (ROS) using the cell permeant, cationic fluorescent dye 2′, 7′-dichlorfluorescein-diacetate (DCFH-DA). Cells were washed with HBSS, and then loaded with 10 μM DCFH-DA and incubated for 30 min in darkness to facilitate fluorophore loading. After being washed 3 times with HBSS in darkness, fluorescence signals were recorded using a fluorescence microscope. Fluorescence images were analyzed using Image-Pro Plus software to index ROS.

### Western blot analysis

Treated cells were lysed with radioimmunoprecipitation assay (RIPA) buffer (Thermo Fisher Scientific, Waltham, USA). The concentration of extracted protein was determined using a Bradford protein assay kit (Bio-Rad Laboratories Inc., Hercules, CA, USA). Then, 20 μg of each protein sample was separated on 10% SDS-PAGE and transferred onto hydrophobic polyvinylidene fluoride (PVDF) membranes. The transferred membranes were then blocked with 5% bovine serum albumin (BSA). The membranes were then sequentially probed with primary antibodies at 4°C overnight and then with the appropriate horseradish peroxidase (HRP)-conjugated secondary antibody. Next, the blots were developed with enhanced chemiluminescence (ECL) reagent (Bio-Rad Laboratories Inc.). Proteins were quantified using Image J software as a proportion of the internal control protein band (β-actin).

### Cytochrome C assay

After the indicated treatment, cells were washed three times with PBS followed by incubation with ice-cold cytosolic extraction buffer (250 mM sucrose, 20 mM Hepes pH 7.4, 10 mM KCl, 1 mM EGTA, 1 mM EDTA, 1 mM MgCl2, 1 mM dithiothreitol, 1 mM phenylmethylsulphonyl fluoride, 1 mM benzamidine, 1 mM pepstatin A, 10 mg/mL leupeptin, and 2 mg/mL aprotonin) for 30 min on ice. Cells were then collected and a glass of Dounce homogenizer was used to homogenize the cell suspensions with a tight-fitting pestle. Then, samples were centrifuged at 2,500 rpm for 10 min at 4°C, and the supernatant was collected and used for another centrifugation at 13,000 rpm for 30 min at 4°C. The yielded cytosolic extract was used to determine the levels of cytochrome C in the cytosol via western blot analysis. Mitochondrial cytochrome C oxidase subunit IV (COX 4) was used as an internal control.

### Determination of mitochondrial ROS

MitoSOX Green from Invitrogen was used to determine mitochondrial ROS according to the manufacturer’s instructions. MitoSOX Green is a live-cell permeant which can be used as a mitochondrial superoxide indicator. Briefly, after the indicated treatment, cells were incubated with 5 mM MitoSOX Green in Hank’s balanced salt solution (HBSS) for 10 min at 37°C. Fluorescent signals were investigated using a fluorescence microscope (with excitation at 510 nm and emission at 580 nm). The average fluorescence intensity of each individual cell was analyzed using Image-Pro Plus software.

### Measurement of Lactate Dehydrogenase (LDH) release

Lactate dehydrogenase (LDH) is an intracellular enzyme, which is released from cells when they suffer injury. Release of LDH from damaged cells was assayed using a commercial kit according to the manufacturer’s instructions. Absorbance measured at 490 nm was used to quantify the amount of LDH. The ratio of LDH activity in the supernatant to total LDH activity was taken as the percentage of cell death in accordance with the manufacturer’s instructions.

### 3-(4, 5-dimethylthiazol-2-yl)-2, 5-diphenyl-tetrazolium bromide (MTT) assay

Cell viability was determined using the 3-(4, 5-dimethylthiazol-2-yl)-2, 5-diphenyl-tetrazolium bromide (MTT) reduction method. Following the indicated treatment, MTT was added into the medium with a final concentration of 1 mg/mL and incubated for 4 h in a CO_2_ incubator at 7°C. The resultant insoluble formazan crystals were dissolved using dimethyl sulfoxide and absorbance was measured at 570 nm using a microtiter plate reader. OD value intensity was used to index cell viability.

### Determination of Adenosine Triphosphate (ATP) levels via bioluminescence assay

Intracellular ATP levels were determined using an adenosine triphosphate (ATP) bioluminescence assay kit following the manual instructions. Cells were lysed with lysis buffer and centrifuged at 10,000×g for 10 min at 4°C. Then, 100 μl of supernatant was mixed with an equal amount of luciferase reagent, which catalyzed the light production from ATP and luciferin. Signals were recorded by a microplate luminometer and used to reflect ATP concentration.

### Terminal deoxynucleotidyl transferase-mediated dUTP biotin nick end labeling (TUNEL) assay

Apoptotic cells were stained using the terminal deoxynucleotidyl transferase-mediated dUTP biotin nick end labeling (TUNEL) method (Wako Pure Chemical, Japan) according to the manufacturer’s instructions. FITC-labeled TUNEL-positive cells were imaged under a fluorescent microscope.

Following the indicated treatment, the nuclear morphology and chromatin condensation in human HCEpiCs were investigated via Hoechst staining. Cells were loaded with 50 μg/ml Hoechst33258 and incubated for 15 min at RT in darkness. Blue fluorescence of nuclear morphology and chromatin condensation was recorded by confocal microscopy.

### Data analysis

All the data in this study are presented as means ± standard error (SE). Statistical differences between groups were analyzed by one-way analysis of variance (ANOVA) followed by Dunnett’s test. P<0.05 was considered as statistically significant.

## Results

**Fig 1:**
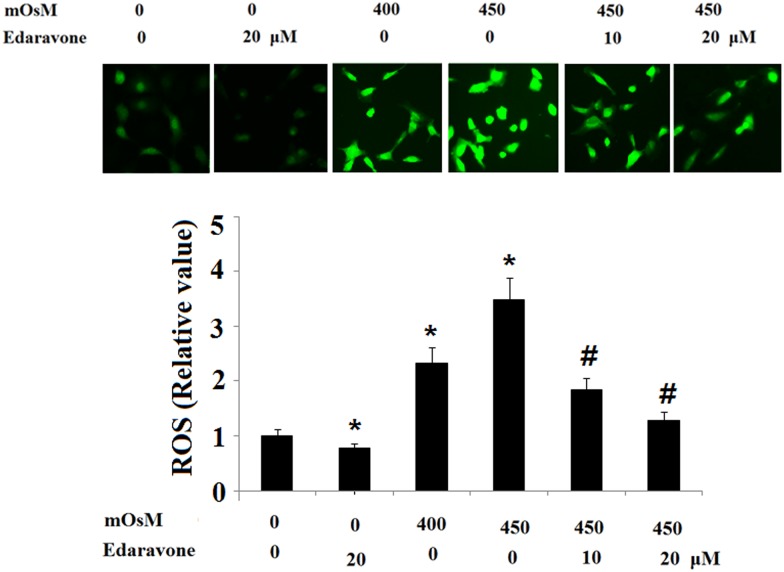
Suppressive effects of edaravone on ROS in primary HCEpiCs exposed to hyperosmotic media. ROS was evaluated via DCFH-DA staining (ANOVA: *, P<0.001 vs. untreated control; #, P<0.001 vs. 450 mOsM treated group).

Edaravone inhibited the production of ROS stimulated by hyperosmolarity in HCEpiCs as evaluated via DCFH-DA assay. The results indicate that fluorescence intensity was markedly increased after cells were treated with hyperosmotic media at 400 and 450 mOsM for 24 h (Fig 1), while prophylactic treatment with 10 μM or 20 μM edaravone attenuated the stimulated ROS production in a dose-dependent manner.

**Fig 2:**
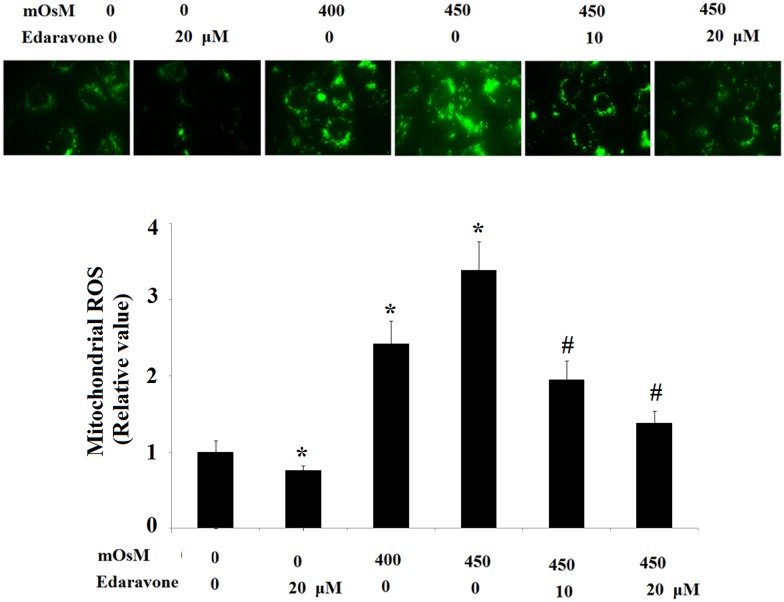
Suppressive effects of edaravone on mitochondrial function at 10 or 20 μM. ROS in primary HCEpiCs exposed to hyperosmotic media (400–450 mOsM), evaluated via MitoSox Red staining (ANOVA: *, P<0.001 vs. untreated control; #, P<0.001 vs. 450 mOsM treated group).

Mitochondria in HCEpiCs are important targets of oxidative damage. We thus investigated the generation of hyperosmolarity-induced mitochondrial oxidant in HCEpiCs using MitoSOX Green probe. Our results indicate that treatment with hyperosmotic media at 400 and 450 mOsM for 24 h significantly increased levels of mitochondrial ROS in HCEpiCs, which was ameliorated by pretreatment with 10 μM or 20 μM edaravone in a dose-dependent manner (Fig 2).

**Fig 3:**
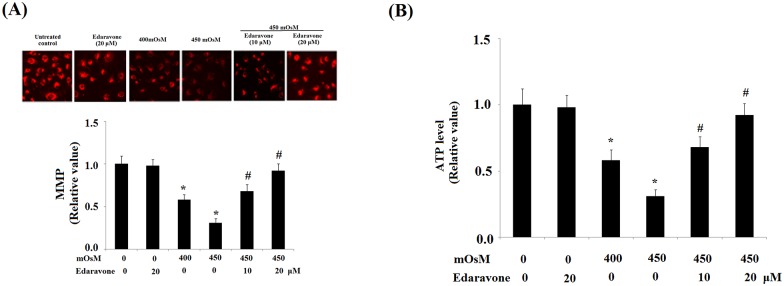
Effects of edaravone on mitochondrial function in primary HCEpiCs exposed to hyperosmotic media. (A) Representative fluorescence photos of mitochondrial membrane potential (MMP) show reduced intracellular MMP in primary HCEpiCs, which was ameliorated by edaravone in a dose-dependent manner; (B) Quantitative results show that exposure to hyperosmotic media (400–450 mOsM) reduced intracellular ATP in HCEpiCs, which was attenuated by edaravone in a dose-dependent manner (ANOVA: *, P<0.001 vs. untreated control; #, P<0.001 vs. 450 mOsM treated group).

We further investigated whether edaravone has an effect on mitochondrial function in HCEpiCs under hyperosmolarity-induced toxicity. We investigated the levels of Ψm in HCEpiCs in an effort to determine mitochondrial function. We examined Ψm using TMRM. It was shown that treatment with hyperosmotic media at 400 and 450 mOsM for 24 h significantly reduced the levels of Ψm (Fig 3A), which could be partially ameliorated by pretreatment with edaravone in a dose-dependent manner. Decreased ATP is an important parameter in determining mitochondrial dysfunction. Following exposure to hyperosmolarity, we found that the level of ATP was significantly reduced in HCEpiCs. However, the impaired production of ATP could be partially attenuated by edaravone treatment (Fig 3B).

**Fig 4:**
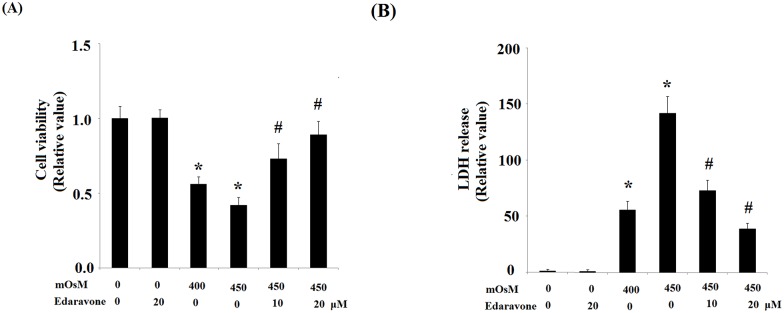
Edaravone prevents cell death induced by exposure to hyperosmotic media (400–450 mOsM). (A) Cell viability was measured by MTT assay; (B) Cell death was measured by LDH release assay. All experiments were repeated at least three times (ANOVA: *, P<0.001 vs. untreated control; #, P<0.001 vs. 450 mOsM treated group).

Cell viability was determined by MTT assay. As shown in Fig 4A, treatment with hyperosmotic media at 400 and 450 mOsM significantly reduced the mean cell viability of HCEpiCs. Pretreatment with edaravone (10 μM or 20μM) had a preventative effects against hyperosmolarity-induced reduction in cell viability. To confirm that edaravone mitigated cell vulnerability to hyperosmolarity, cellular toxicity levels were determined using an LDH assay. As expected, edaravone pretreatment significantly reduced the hyperosmolarity-induced release of LDH in HCEpiCs in a dose-dependent manner (Fig 4B).

**Fig 5:**
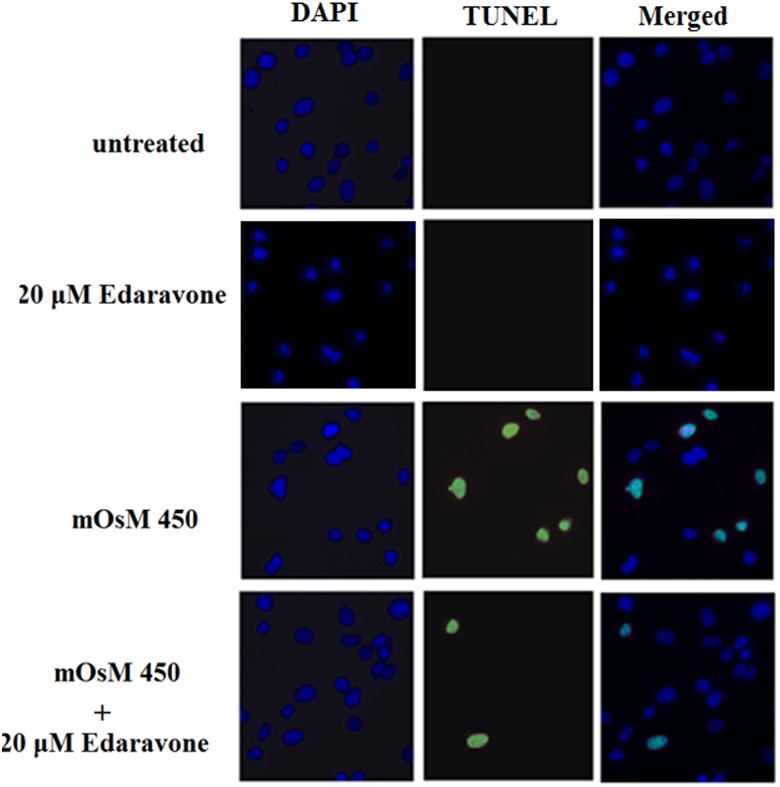
Effects of edaravone on apoptosis in primary HCEpiCs exposed to hyperosmotic media (400–450 mOsM). Apoptotic cells were stained using a TUNEL Assay Kit. Nuclear DNA was stained with DAPI.

To determine whether edaravone could have a direct effect on hyperosmolarity-induced apoptosis, patterns of cell apoptosis was measured via TUNEL staining following treatment with hyperosmotic media at 400 and 450 mOsM for 24 h. As shown in Fig 5, few TUNEL-positive cells were found in normal HCEpiCs. By contrast, the ratio of TUNEL-positive cells was notably increased in HCEpiCs treated with 450 mOsM. However, pretreatment with edaravone could ameliorate the number of TUNEL-positive cells under hyperosmolarity.

**Fig 6:**
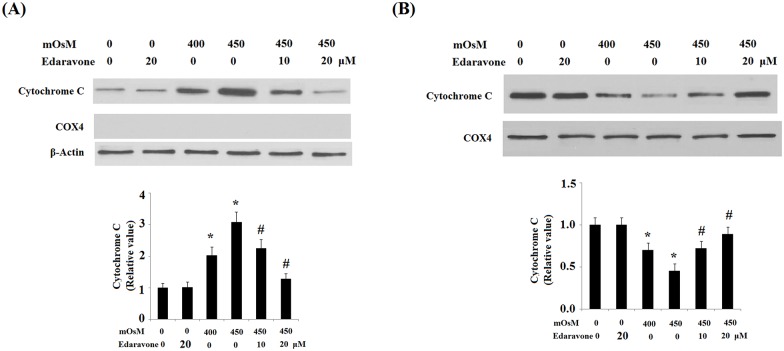
Effects of edaravone on cytosol cytochrome C release as measured by western blot analysis. Results indicate that edaravone treatment attenuates hyperosmotic exposure (400–450 mOsM)-induced release of cytochrome C. (A) Cytosolic fractions; (B) Mitochondrial fractions (ANOVA:*, P<0.001 vs. non-treatment control; #, P<0.001 vs. 450 mOsM treated group).

Impaired Ψm is associated with translocation of the apoptogenic protein cytochrome C into the cytoplasm and the subsequent cleavage and activation of caspase-3 [12]. Therefore, we examined the effects of edaravone on the release of cytochrome C. As shown in Fig 6A, cytochrome C in cytosol was significantly increased in HCEpiCs after treatment with hyperosmotic media at 400 and 450 mOsM for 24 h, which was significantly ameliorated by pretreatment with edaravone. COX 4, another mitochondrial protein, was used to confirm that the purified cytosolic fractions were not contaminated with mitochondrial proteins. In contrast, the level of cytochrome C in mitochondria was significantly reduced after treatment with hyperosmotic media, which was markedly blunted by pretreatment with edaravone.

**Fig 7:**
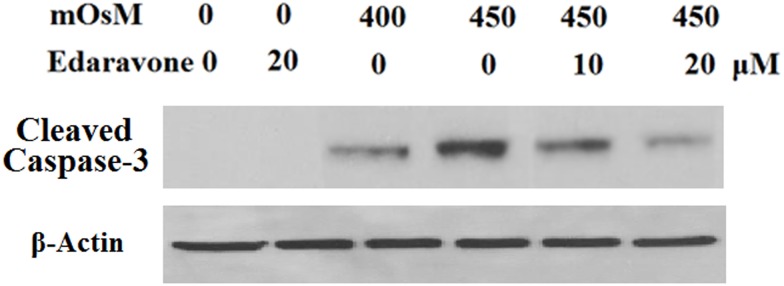
Effects of edaravone on cleaved caspase-3 as measured by western blot analysis. Edaravone treatment inhibits the increase in cleaved caspase-3 induced by hyperosmotic exposure (400–450 mOsM) (ANOVA:*, P<0.001 vs. non-treatment control; #, P<0.001 vs. 450 mOsM treated group).

**Fig 8:**
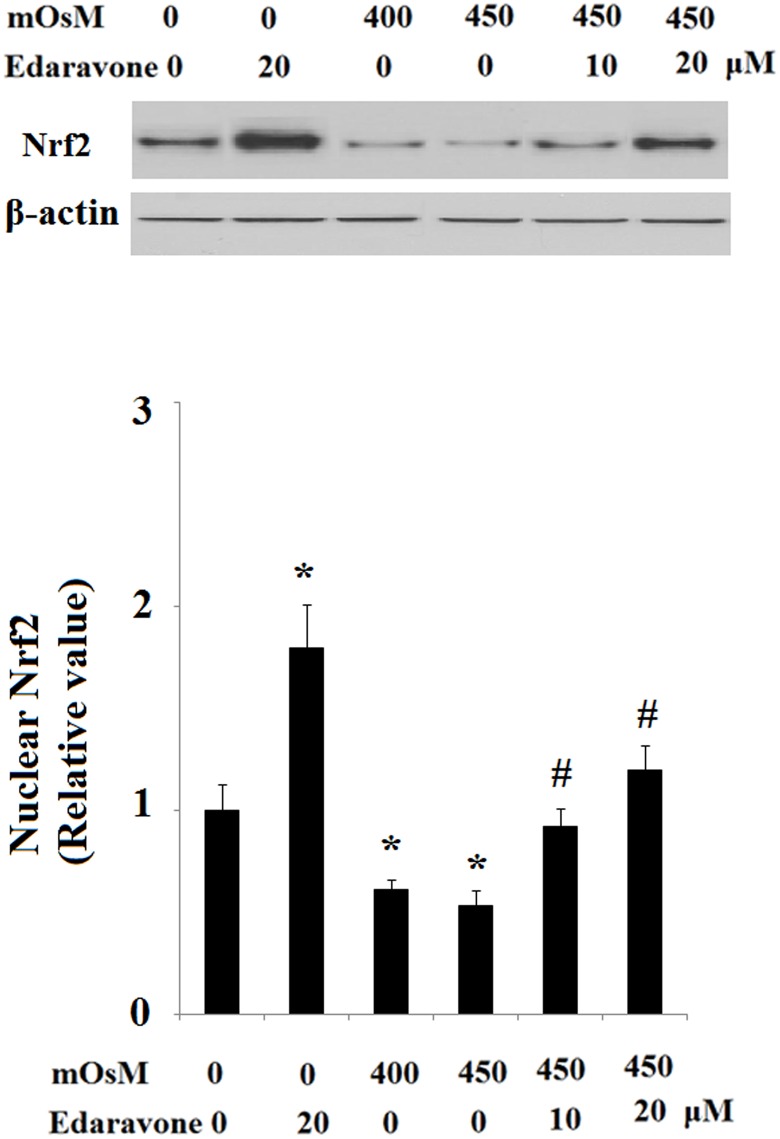
Effects of edaravone on the expression of Nrf2. Levels of Nrf2 in whole cell extracts were determined by the western blot analysis (ANOVA:*, P<0.001 vs. non-treatment control; #, P<0.001 vs. 450 mOsM treated group).

**Fig 9:**
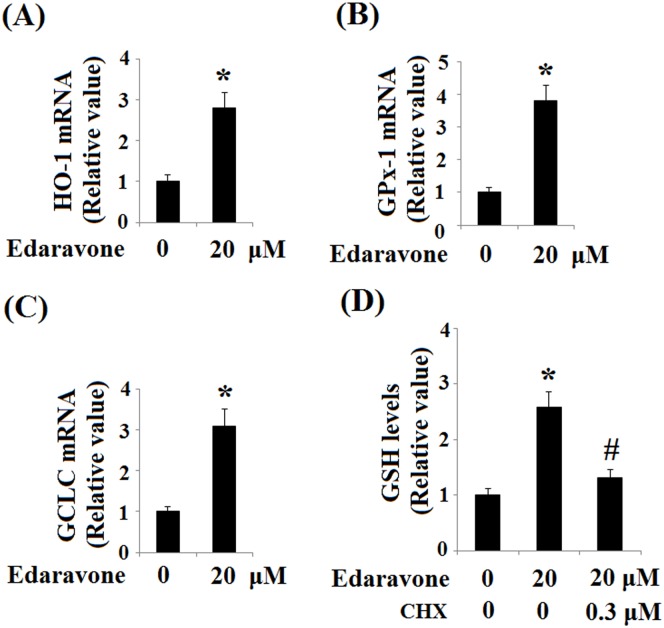
Effects of edaravone on Nrf2 target gene expression in primary HCEpiCs. Cells were treated with edaravone for 24 h. Gene expression was determined by real-time PCR analysis. (A) HO-1 mRNA; (B) GPx-1 mRNA; (C) GCLC mRNA; (D) GSH levels (ANOVA:*, P<0.001 vs. non-treatment control; #, P<0.001 vs. 450 mOsM treated group).

In addition, it was shown that hyperosmolarity treatment leads to cleavage of caspase-3 in HCEpiCs and that the activation of caspase-3 is significantly suppressed by pretreatment with edaravone (Fig 7). To further define some of the signaling systems activated by hyperosmolarity that could mediate the protective effects of edaravone, the expression of transcriptional factor Nrf2 was assessed. As shown in Fig 8, western blot results revealed that edaravone treatment significantly elevated the expression of Nrf2. However, hyperosmolarity markedly reduced Nrf2 expression after 24 h of exposure in HCEpiCs, which could be ameliorated by administration of edaravone. In order to confirm the effects of edaravone treatment on Nrf2 activation, we assessed the expression of some of its target genes at the mRNA level using real-time PCR: HO-1, GPx-1 and GCLC. As shown in Fig 9A–9C, edaravone treatment leads to an increase in the mRNA levels of Nrf2-target genes after 24 h. Moreover, to determine whether edaravone modulates anti-oxidant mechanisms through a genomic effect dependent on Nrf2, we determined the level of GSH in the presence of CHX, a classical protein synthesis inhibitor. As shown in Fig 9D, co-treatment with 0.3 μM of CHX opposed the edaravone-induced upregulation of GSH in HCEpiCs. Taken together, these results support our hypothesis that edaravone can improve the cellular antioxidant response by inducing the nuclear transcriptional factor Nrf2.

## Discussion

Tear hyperosmolarity plays a central role in ocular surface damage related to dry eye [13], and oxidative stress is an important contributing factor to hyperosmolarity. A recent study showed that hyperosmolarity induces oxidative stress in HCECs by stimulating the generation of ROS and suppressing protein levels of the antioxidant enzymes SODl and GPXl, which in turn causes a significant increase in cellular oxidative damage biomarkers resulting from lipid peroxidation of cell membranes (HNE and MDA) and in mitochondrial DNA (8-OHdG and aconitase-2). In addition, both *in vivo* and *in vitro* studies have shown that hyperosmotic stress induces the expression and production of proinflammatory cytokines in cells on the ocular surface [14]. Notably, hyperosmolarity-induced ocular surface cell death is a key mitochondria-mediated event in inflammatory eye diseases. Hyperosmolarity has been reported to induce mitochondrial depolarization and stimulate mitochondrial cell death in human corneal epithelial (HCE-T) cells [15]. Countless types of natural and synthesized chemical agents have been tested for the prevention of hyperosmolar stress on corneal epithelial cells. The key finding of the current study is that edaravone, a powerful free radical scavenger, inhibited hyperosmolarity-induced oxidative stress, mitochondrial dysfunction, and apoptosis.

With the exception of treatment for stroke, the neuroprotective effects of edaravone have been verified in a number of diseases in which free radicals contribute to cell death, including both neurological and non-neurological diseases. However, reports on the protective effects of edaravone in dry eye disease are few. To the best of our knowledge, this is the first report to date on the protective effects of edaravone against hyperosmolarity-induced toxicity. This effect is achieved by the regulation of ROS levels, mitochondrial function, and Nrf2 expression. As a potent free radical scavenger, edaravone exerts a wide range of antioxidant activities on ROS by quenching hydroxyl radicals (•OH) and exerting an inhibitory effect on peroxynitrite (ONOO−) [9, 15]. Edaravone quenches •OH and inhibits both •OH-dependent and •OH-independent lipid peroxidation. Mitochondrial dysfunction is implicated in the pathogenesis of dry eyes. Hyperosmolarity leads to mitochondrial depolarization. Decreased Ψm has been implicated as a factor in impaired mitochondrial function [16]. A decrease in Ψm is followed by an intense burst of ROS production which leads to mitochondrial disruption, inhibition of the mitochondrial respiratory chain, a reduction in ATP synthesis, and cell death [17]. Edaravone has been reported to protect lung isolated mitochondria against ROS generation and mitochondrial dysfunction induced by the toxin Paraquat (PQ) [18]. Notably, a previous study found that edaravone improves mitochondrial function and reduces apoptosis by modulating mitochondria-dependent apoptosis pathways [19].. These data are consistent with our findings.

The antioxidant effects of edaravone have been studied to a great extent. The Nrf2 pathway is involved in the cellular response to oxidative stress and leads to the transcription of several antioxidant genes. The current study shows significant up regulation in Nrf2 levels during edaravone treatment. This enhanced oxidative stress induced by hyperosmolarity may be associated with a decline in Nrf2, the master regulator of antioxidant enzymes. Similarly, edaravone was recently reported to enhance Nrf2 and inhibit oxidative stress [20]. Targeting the Nrf2 pathway has become a potential therapeutic avenue for dry eye diseases. The Nrf2/antioxidant response element (ARE) pathway is a critical signaling pathway regulating antioxidants and phase II detoxification enzymes, such as hemeoxygenase 1 (HO-1), NAD(P)H quinoneoxido reductase 1 (NQO1), glutathione peroxidase (GPx), glutamate cysteine ligase (GCLC) and thioredoxin reductase 1 (TrxR1) [21]. These findings suggest that the protective effects of edaravone against hyperosmolarity are a result of its anti-oxidant capacity. Further investigation is necessary and exploration of the protective effects and mechanism of edaravone on dry eye using *in vitro*, *ex-vivo* and/or *in vivo* dry eye models will make for an interesting line of research.

## Conclusions

In the present work, we demonstrated the protective action of edaravone in hyperosmotic culture models of human corneal epithelial cells (HCECs). The drug partly reversed alterations in the generation of ROS and mitochondrial oxidative stress induced by hyperosmolarity, and also improved mitochondrial dysfunction. In addition, edaravone treatment attenuated hyperosmolarity-induced cell death and apoptosis. Mechanically, edaravone augmented the expression of Nrf2 and its target genes, such as HO-1, GPx-1 and GCLC. These findings strongly suggest that edaravone could be a potential candidate for the treatment of hyperosmolarity-induced eye diseases.
